# Personal microbiome analysis improves student engagement and interest in Immunology, Molecular Biology, and Genomics undergraduate courses

**DOI:** 10.1371/journal.pone.0193696

**Published:** 2018-04-11

**Authors:** K. Scott Weber, Laura C. Bridgewater, Jamie L. Jensen, Donald P. Breakwell, Brent L. Nielsen, Steven M. Johnson

**Affiliations:** 1 Department of Microbiology and Molecular Biology, Brigham Young University, Provo, Utah, United States of America; 2 Department of Biology, Brigham Young University, Provo, Utah, United States of America; University of Illinois at Urbana-Champaign, UNITED STATES

## Abstract

A critical area of emphasis for science educators is the identification of effective means of teaching and engaging undergraduate students. Personal microbiome analysis is a means of identifying the microbial communities found on or in our body. We hypothesized the use of personal microbiome analysis in the classroom could improve science education by making courses more applied and engaging for undergraduate students. We determined to test this prediction in three Brigham Young University undergraduate courses: Immunology, Advanced Molecular Biology Laboratory, and Genomics. These three courses have a two-week microbiome unit and students during the 2016 semester students could submit their own personal microbiome kit or use the demo data, whereas during the 2017 semester students were given access to microbiome data from an anonymous individual. The students were surveyed before, during, and after the human microbiome unit to determine whether analyzing their own personal microbiome data, compared to analyzing demo microbiome data, impacted student engagement and interest. We found that personal microbiome analysis significantly enhanced the engagement and interest of students while completing microbiome assignments, the self-reported time students spent researching the microbiome during the two week microbiome unit, and the attitudes of students regarding the course overall. Thus, we found that integrating personal microbiome analysis in the classroom was a powerful means of improving student engagement and interest in undergraduate science courses.

## Introduction

One area of rapid scientific progress and interest is the human microbiome. All organisms, including humans, exist within a sea of microbial communities (termed the ‘microbiome’) [1]. The human gut microbiome consists of trillions of microorganisms and it is increasingly clear that the diversity and composition of this community of microorganisms has a profound effect on human health and disease [1–3]. Recent pedagogy studies have found integration of current technologies and issues into the classroom can improve the relevance of the course and student learning [4–7]. We determined to see if integration of personal microbiome analysis into the classroom would be an effective means of improving student interest and engagement in undergraduate science classes.

Our understanding of the importance of the human microbiome is still emerging as technologies and analysis improve and DNA sequencing costs fall. Over the past two decades there have been numerous studies documenting the role of the microbiome on human physiology, metabolism, immunity, and the development of associated diseases (e.g. cardiovascular, gastrointestinal, allergic, and autoimmune diseases) [8–10]. The Human Microbiome Project (completed in 2012) sequenced the metagenomes of thousands of microbes from numerous sites on and in the human body of several hundred people, providing important insights into the microbiomes associated with health and disease [11–13]. Clinical trials are currently being conducted using gut microbiota as potential preventative or therapeutic agents for antibiotic-resistant pathogens such as *Clostridium difficile* infections, treating pancreatic islet autoimmunity, and to inoculate infants and children against malnourishment [14–17].

Personal microbiome companies offer kits that allow consumers to independently obtain phylogenetic information about the composition of the microbiome at various sites like the intestine, mouth, nose, and skin. The ethics of what can be learned from these kits (e.g. how it affects our understanding of personal identity, normalcy, privacy, and property) and how these data should be used can be a timely and applicable classroom discussion topic [18–23]. Analyzing data with personal relevance may increase student motivation to understand the results since these results could directly impact their lives. Ethical considerations of microbiome analysis include wrestling with understanding what our microbiome profile means in terms of personal distinguishing characteristics. As we learn more about how our microbiome can be altered by our life history and experience (genetics, nutrition, interpersonal interactions, etc), understanding these data may increasingly affect our identity and sense of normalcy compared to others. Thus, access to personal microbiome data may enable classroom discussions that are more relevant to the students.

Stanford University researchers have reported inclusion of personal genome analysis in medical school instruction improved student engagement and learning based off of self-reported responses and significantly greater improvement in a personal-genomics-knowledge quiz compared to the control group [24]. Using similar methods, we previously found that anticipation of personal genomics analysis improved undergraduate student interest and personal-genomics quiz scores, even though these undergraduate students did not actually analyze their data until after the course was complete [25]. Based off of these findings, we hypothesized that specific homework assignments and online modules walking students through analysis of their own microbiome data would be an engaging and practical means of improving classroom learning. We specifically wanted to test if the analysis of this personalized microbiome data would provide added incentive for undergraduate students learning immunology, genomics, and molecular biology. This premise was based on motivational theory, specifically personal relevance, and the idea that students would be motivated by the idea of analyzing their own microbiome data due to the personal value in the task and that this would result in improved engagement [26]. Researchers have found that integration of personally relevant content helps to motivate students to engage more in the learning experience [27, 28].

The theoretical rationale for our hypothesis is based in motivational theory, specifically the idea of personal relevance. There are several ways of framing personal relevance within motivational theory. We define personal relevance as being information that is personally significant to the learner. Using the four-phase model of interest development as a motivational framework [29], providing personal microbiome data may serve as a trigger for situational interest and may increase students’ cognitive energies during the activity. Using the expectancy-value model of motivation [30], the personal relevance of their own data may serve a utility value in that it may help students obtain a personal health and/or fitness goal. In contrast to our earlier work in which students anticipated analyzing their own static personal genomic data (i.e. genomic data does not change) [25], a students’ microbiome is dynamic based on changes in their lifestyle. Thus, obtaining this information may give students personal utility value as they seek to improve their own health. Research shows that individuals are more motivated to learn when they see the usefulness of the information [31].

Using the self-determination theory [32–34] as a framework for motivation, we hypothesize that obtaining one’s own data would shift motivation from extrinsic, where students are only completing assignments for the sake of obtaining a grade, to more intrinsic, where students are seeking out the information because they genuinely want to know. Research has shown that intrinsic motivation leads to higher achievement [35, 36]. As Priniski et al. pointed out [37], each of these models for motivation based on personal relevance overlap one another and add value to the way in which we conceptualize the influence of relevance on motivation and subsequent learning. We would place this current task on Priniski’s newly proposed continuum between personal usefulness and the strongest motivator, that of personal identification. Using this framework, we hypothesize that access to their own microbiome data provides students with personal relevance, which leads to increased motivation by providing situational interest [29], utility value [30], and intrinsic motivation [32–34]. We predict that this increased motivation will lead to greater cognitive expenditure, engagement, and learning.

Personal genomics is an established field in which the data for each individual is fixed, whereas personal microbiome analysis is a newer field based off of personal data that can fluctuate depending on diet, lifestyle, and use of antibiotics. We wondered if these differences in the nature of this personal data would alter the motivation of undergraduate students to learn so they could better analyze their own data. In this study, we examined whether analysis of personal microbiome data improves student engagement and interest in three undergraduate science courses at Brigham Young University (Advanced Molecular Biology Laboratory, Immunology, and Genomics). Of note, we found that students who analyzed their personal microbiome data reported significantly enhanced engagement and interest, increased self-reported time spent researching microbiome material, and significantly improved attitudes regarding the course overall.

## Materials and methods

### Subjects

Subjects for this study were undergraduate students enrolled in one of three undergraduate science courses at Brigham Young University (Advanced Molecular Biology Laboratory, Immunology, or Genomics) during the Winter 2016 and 2017 semesters. Students enrolled in these three courses had all previously completed an introductory molecular biology course. During the 2016 semester students could submit their own personal microbiome kit or use the demo data, whereas during the 2017 semester students were given access to microbiome data from an anonymous individual. In 2016 students were given the option to use a five-site uBiome kit (https://www.uBiome.com) which allowed for sampling of the nose, mouth, skin, gut (stool), and genitals (or alternative site). The five-site microbiome kits were purchased using funds from a Brigham Young University Teaching Enhancement Grant incurring no cost to the students. Sixty-five students completed both the pre- and post-surveys and received personal microbiome kits; eighty students completed both the pre- and post-surveys and used demo data. Students who did not complete both the pre- and post-surveys were not included in the data analysis (Forty students total; fifteen in 2016 and 25 in 2017). The survey contained a written consent form that was documented in each completed survey and the Brigham Young University Institutional Review Board approved the study methodology (Study # E16071).

### Microbiome analysis

The three courses in this study had a two-week focus on the microbiome at the end of the semester. Before starting the microbiome unit, students were asked to read articles on what is known and unknown regarding the microbiome and complete a homework assignment on the reading. Students in the 2016 courses were given the option to decide whether or not they wanted a personal microbiome kit (sixty-five students opted to receive a kit and five students opted to not receive a kit). A classroom discussion reviewing the same material of what was known and what was not known regarding current analysis of the microbiome was led by the instructors (K.S.W. for Immunology and Advanced Molecular Biology laboratory and S.M.J. for Genomics; discussions led by the same instructor for these classes both years) [38, 39]. Students in the 2016 courses who opted to receive a microbiome kit collected their own samples, sent them for testing, and were the only people with access to their microbiome data.

During the two-week microbiome unit, a classroom discussion on how the data were obtained and what could and could not be concluded from these data was led by the instructors (K.S.W. and S.M.J). The instructors also demonstrated how to analyze microbiome data using the demo data from an anonymous individual. Students in all three courses were asked to complete an online module with four assignments that required analysis of their data or the demo data provided.

### Survey instrument

At the start and conclusion of the two-week microbiome emphasis, a survey was administered to measure the student attitudes, understanding of personal microbiome analysis, and the classroom learning experience. Assessment of student attitudes was measured by agreement with statements on a 5-point Likert scale (1 = strongly disagree, 2 = disagree, 3 = neither agree or disagree, 4 = agree, 5 = strongly agree), and results are displayed as the mean. We also assessed student behavior and attitudes as they answered questions while completing four online modules. In the pre-survey we assessed the biological training of the students to control for potential group non-equivalence using a 17-question multiple-choice quiz (S1 Fig) taken from the 24-item Introductory Molecular and Cellular Biology Assessment (IMCA) [40]. The IMCA is a published biology concept inventory that includes questions on molecular biology, cellular biology, genetics, and gene expression. Validity and reliability of the full IMCA has been established [40]. In the post-survey, we assessed student understanding of the microbiome by scoring a 10-question multiple-choice quiz created by researchers based on the learning outcomes of the course. Expert validity was established by a team of researchers familiar with the content and the project. Several iterative rounds were made to match the test to course learning outcomes as agreed upon by the researchers. The responses and scores of students given the personal microbiome kits (2016) were compared to students who were not given the kits (2017) but had access to the demo microbiome profile of an anonymous individual.

### Data analysis

Data analysis was performed on responses from students who had completed both the pre- and post-surveys and had not previously completed microbiome testing and analysis. Any students enrolled in more than one of the three courses in the study (either in 2016 or 2017) were only counted once (Data for a student enrolled in two of these courses was included with the class in which they did the survey first). A 5-point Likert scale was used to access student attitudes and data comparisons between groups (kit and no kit) were performed using a Mann-Whitney U-test. For categorical survey questions, statistical analysis of student answers was performed using a chi-square test and all values are shown as stacked bars (percentages of the categories chosen). Statistics were performed using Prism 7 software (GraphPad).

## Results

These students were all undergraduates taking either the Advanced Molecular Biology Laboratory, Immunology, or Genomics courses at Brigham Young University during winter semester of 2016 or winter semester of 2017. For the online module Sixty-five students received personal microbiome kits and eighty students used demo data. Only students who completed both the pre- and post-surveys were included in this study. For the post-survey students waiting to receive their data were grouped with the no kit cohort.

### Basic biology preparedness of students in study

**Fig 1:**
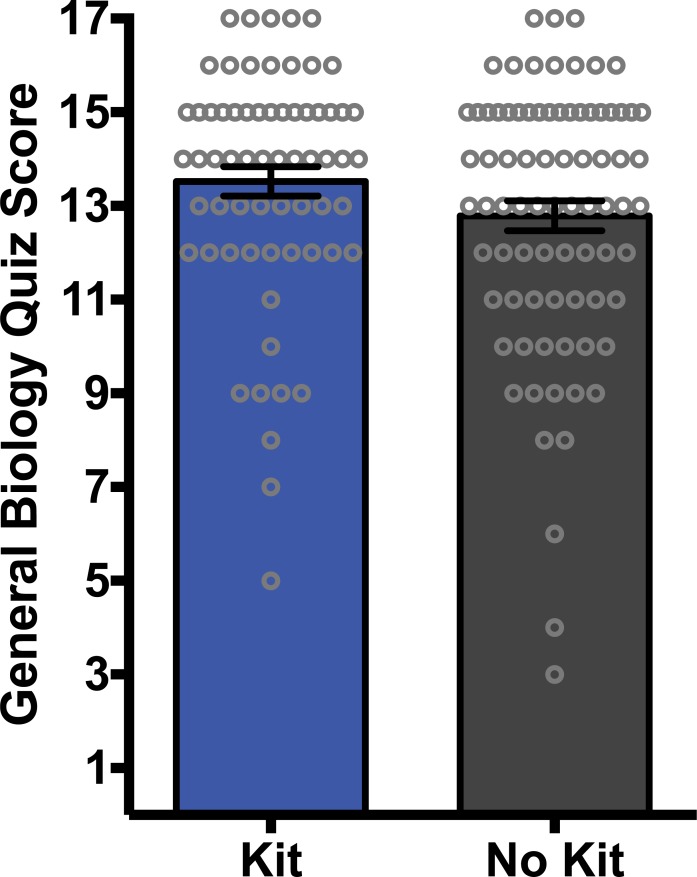
Student basic biology score on pre-survey quiz. Quiz score on the 17-question basic biology quiz evaluating the prior biological preparation of students in the classes. There were no significant differences on the biology-quiz scores between the students receiving a kit and those who did not receive a kit. Statistical analysis performed using a Mann-Whitney U-test and all values are mean +/- SEM with n = 65 for kit (blue) and n = 80 for no kit (black). Grey circles represent individual student scores and show the overall score distribution for each group.

Before starting the study and to test for group non-equivalence, we wanted to determine whether the groups being compared had significantly different levels of basic biology background prior to the microbiome unit in our classes. As part of the microbiome pre-survey students were given a 17-question quiz on basic biological topics to evaluate the prior biological preparation of students in the classes (S1 Fig). There were no significant differences on the basic biology quiz between the students who received the microbiome kits compared to those who did not (Fig 1; p = 0.11).

### Online module assignment #1 –health effects of most abundant microbiome phylum

**Fig 2:**
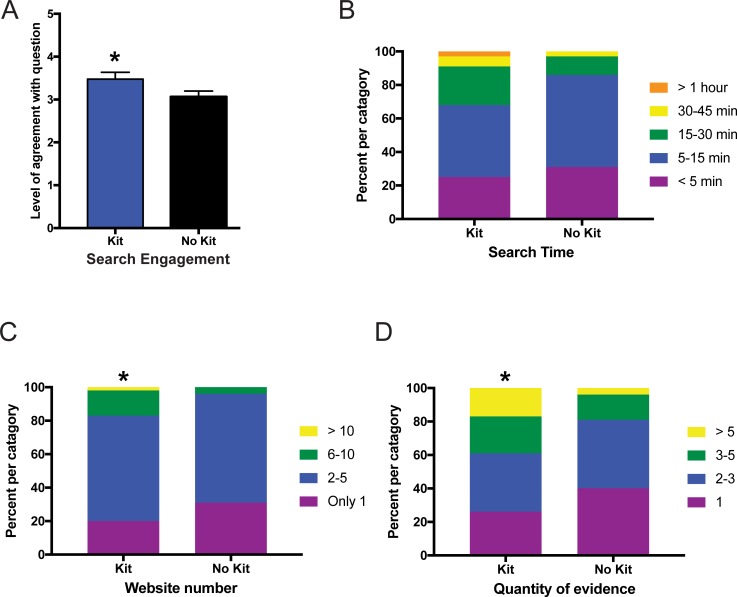
Online microbiome module assignment #1 evaluating health effects of the most abundant microbiome phylum. Measurement of student responses regarding their agreement with questions about the online microbiome module assignment #1. Students who were given a kit evaluated their own microbiome data whereas those without a kit evaluated demo data. (A) Level of agreement for how engaged the students were during an internet search examining how bacterial phylum influences health. Statistical analysis for (A) performed using a Mann-Whitney U-test and all values are mean ± SEM with n = 65 for kit (blue) and n = 80 for no kit (black) (* = p<0.05). (B) Self-reported time spent searching showed equivalent levels of time between groups whereas the students analyzing their own microbiome data had significantly higher number of website visits (C) and quantity of evidence collected (D). Statistical analysis for (B-D) were performed using a chi-square test and all values are shown as stacked bars (percentages of the categories chosen) with n = 65 for kit and n = 80 for no kit (* = p<0.05).

After taking the pre-survey and completing a reading assignment and homework on the basics of the microbiome, students were assigned an online module as homework that included four different research tasks using their own or the demo microbiome data (S2 Fig). The first assignment asked the students to determine their most abundant microbiome phylum and then hypothesize the function of this microbe in the gut. They performed an online search and researched websites describing the health effects of this microbe. Students then reported their search engagement, search time, number of websites visited, and the quantity of evidence they found for or against their original hypothesis of the microbe health function. Students evaluating their own microbiome data reported significantly higher levels of search engagement (Fig 2A; p<0.05), spent equivalent time searching for evidence (Fig 2B; p = 0.08), visited more websites (Fig 2C; p<0.05), and collected more evidence (Fig 2D; p<0.05) than those evaluating demo data.

### Online module assignment #2 –microbiome comparison to lifestyle subgroups

**Fig 3:**
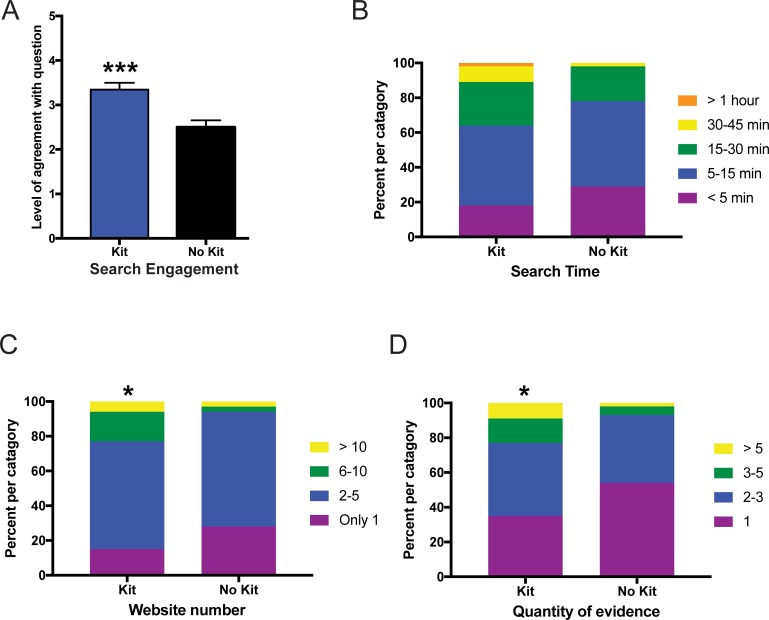
Online microbiome module assignment #2 comparing data to 14 subgroups with different dietary lifestyles. Measurement of student responses regarding their agreement with questions about the online microbiome module assignment #2. Students who were given a kit evaluated their own microbiome data whereas those without a kit evaluated demo data. (A) Level of agreement for how engaged the students were during an internet search regarding diet and lifestyle and microbiome profiles. Statistical analysis for (A) performed using a Mann-Whitney U-test and all values are mean ± SEM with n = 65 for kit (blue) and n = 80 for no kit (black) (*** = p<0.001). (B) Self-reported time spent searching showed equivalent levels of time between groups whereas the students analyzing their own microbiome data had significantly higher number of website visits (C) and quantity of evidence collected (D). Statistical analysis for (B-D) were performed using a chi-square test and all values are shown as stacked bars (percentages of the categories chosen) with n = 65 for kit and n = 80 for no kit (* = p<0.05).

The second assignment asked the students to compare their overall microbiome diversity to a database containing data from 14 sub-groups of people with different lifestyles (i.e. omnivore, vegans, antibiotics, weight loss, gluten free, heavy drinkers, low carb, etc.), determine which group their microbiome diversity most closely resembled, and see if this made sense based on their lifestyle (S2 Fig). They performed an online search and researched websites describing microbiome diversity and lifestyle. Students then reported their search engagement, search time, number of websites visited, and the quantity of evidence they found for or against whether the similarities and differences between their data and the lifestyle sub-groups made sense based on their own lifestyle. Students evaluating their own microbiome data reported significantly higher levels of search engagement (Fig 3A; p<0.001), spent equivalent time searching for evidence (Fig 3B; p = 0.18), visited more websites (Fig 3C; p<0.05), and collected more evidence (Fig 3D; p<0.05) than those evaluating demo data.

### Online module assignment #3 –reliability of microbiome metabolic data

**Fig 4:**
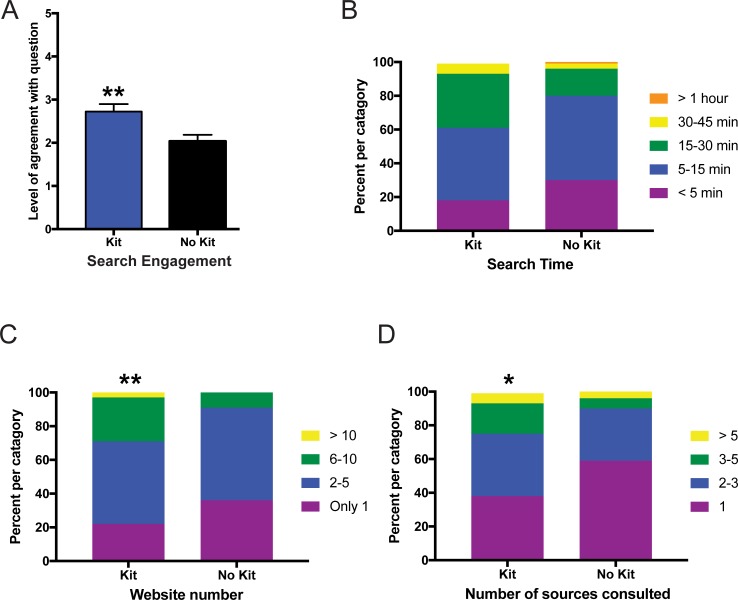
Online microbiome module assignment #3 evaluating the reliability of microbiome metabolic data. Measurement of student responses regarding their agreement with questions regarding online microbiome module assignment #3. Students who were given a kit evaluated their own microbiome data whereas those without a kit evaluated demo data. (A) Level of agreement for how engaged the students were during an internet search regarding the validity of inferring metabolic function from the microbiome profile data. Statistical analysis for (A) performed using a Mann-Whitney U-test and all values are mean ± SEM with n = 65 for kit (blue) and n = 80 for no kit (black) (** = p<0.01). (B) Self-reported time spent searching showed equivalent levels of time between groups whereas the students analyzing their own microbiome data had significantly higher number of website visits (C) and number of sources consulted (D). Statistical analysis for (B-D) were performed using a chi-square test and all values are shown as stacked bars (percentages of the categories chosen) with n = 65 for kit and n = 80 for no kit (* = p<0.05; ** = p<0.01).

The third assignment asked the students to evaluate the reliability of the microbiome metabolic conclusions inferred from the microbiome analysis (S2 Fig). While sequence data enabled identification of different microbes, the entire genomes of all of the microbes have not been completed, and the analysis performed did not look at gene expression. Students were asked to evaluate the metabolic data and they performed an online search and researched websites describing methods of inferring function from taxonomic sequencing data. Students then reported their search engagement, search time, number of websites visited. In case the websites they visited had similar sources, students were also asked for the number of sources they consulted regarding the metabolic conclusions from sequence data. Students evaluating their own microbiome data reported significantly higher levels of search engagement (Fig 4A; p<0.01), spent equivalent time searching for evidence (p = 0.08) (Fig 4B), visited more websites (p<0.01) (Fig 4C), and consulted more sources (p<0.05) (Fig 4D) than those evaluating demo data.

### Online module assignment #4 –taxonomy of most abundant microbe species

**Fig 5:**
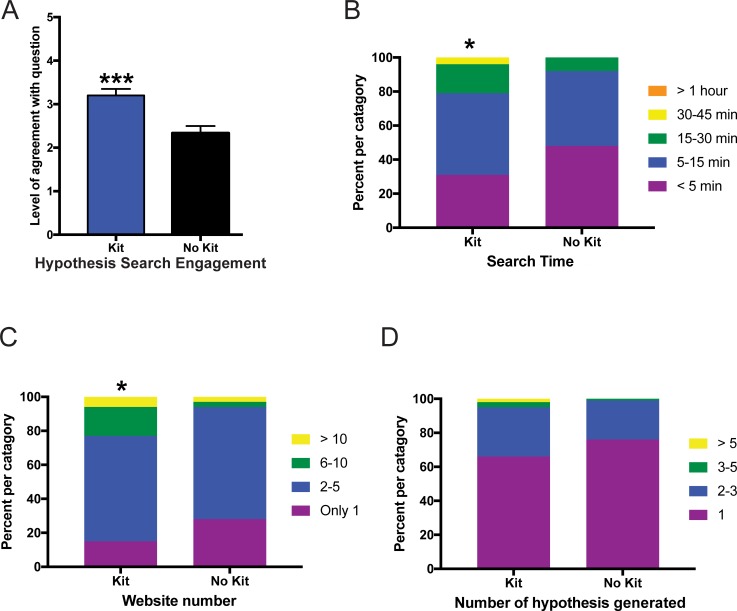
Online microbiome module assignment #4 determining the taxonomy of the most abundant species in the microbiome data. Quantification of student responses regarding their agreement with questions regarding online microbiome module assignment #4. Students who were given a kit evaluated their own microbiome data whereas those without a kit evaluated demo data. (A) Level of agreement for how engaged the students were during an internet search regarding how a microbial species in the sample could affect health. Statistical analysis for (A) performed using a Mann-Whitney U-test and all values are mean ± SEM with n = 65 for kit (blue) and n = 80 for no kit (black) (*** = p<0.001). (B) Students analyzing their own microbiome data had significantly higher self-reported search time and number of website visits (C) whereas the quantity of evidence collected was equivalent between groups (D). Statistical analysis for (B-D) were performed using a chi-square test and all values are shown as stacked bars (percentages of the categories chosen) with n = 65 for kit and n = 80 for no kit (* = p<0.05).

The fourth assignment asked the students to evaluate the taxonomy of the most abundant microbe in the microbiome and generate one hypotheses as to why this specific species might be the most abundant (S2 Fig). They performed an online search and researched websites regarding the most abundant species and then reported their search engagement, search time, number of websites visited, and the number of hypotheses they generated regarding why this species might be the most abundant. While students were only asked to come up with one hypothesis, they were given the option to report how many they actually came up with to see if their personal interest resulted in them thinking more in depth than just the required assignment. Students evaluating their own microbiome data reported significantly higher levels of search engagement (Fig 5A; p<0.001), spent more time searching for evidence (Fig 5B; p<0.05), and visited more websites (Fig 5C; p<0.05) than those evaluating demo data. They did not generate more hypotheses regarding the reason why a bacterial species might be more abundant than those evaluating demo data (Fig 5D; p = 0.40).

### Overall student experience with the online microbiome module

**Fig 6:**
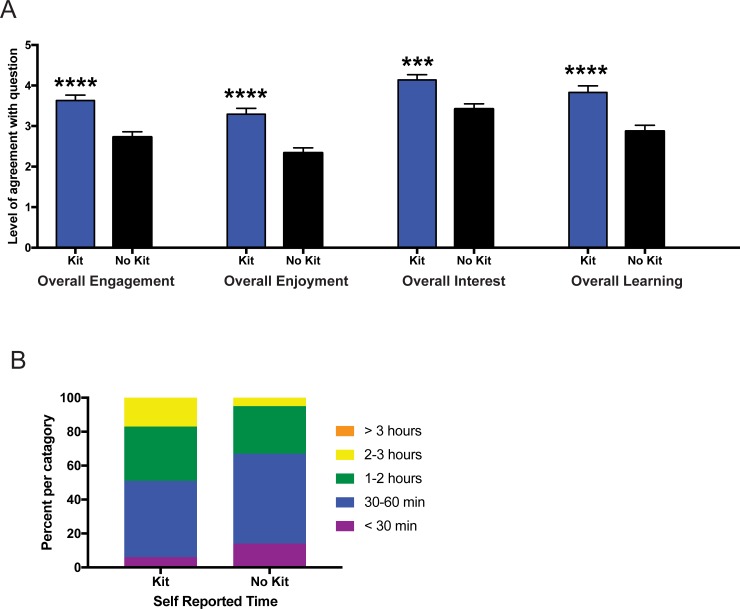
Overall experience with the online microbiome module. Measurement of student responses regarding their agreement with questions regarding their experience overall with the online microbiome module. (A) Students who were given a kit (i.e. evaluated their own microbiome data) reported significantly higher levels of engagement, enjoyment, interest, and learning. Statistical analysis for (A) was performed using a Mann-Whitney U-test and all values are mean ± SEM with n = 65 for kit (blue) and n = 80 for no kit (black) (*** = p<0.001; **** = p<0.0001). (B) Students self-reported equivalent overall time spent in regards to their experience with the online microbiome modules. Statistical analysis for (B) was performed using a chi-square test and all values are shown as stacked bars (percentages of the categories chosen) with n = 65 for kit and n = 80 for no kit.

At the end of the online assignment (S2 Fig), students were asked to evaluate their overall experience with the online microbiome module using a 5-point Likert scale. Students who were given kits and evaluated their own data reported significantly higher levels of engagement (p<0.0001), enjoyment (p<0.0001), interest (p<0.001), and learning (p<0.0001) compared to the students evaluating demo data (Fig 6A). While there was a trend towards students with a kit to spend more time working on the microbiome module, the self-reported time spent on the microbiome module was statistically equivalent (p = 0.07) with students evaluating demo data (Fig 6B).

### Survey on students attitudes towards microbiome testing after two-week focus on the microbiome

**Fig 7:**
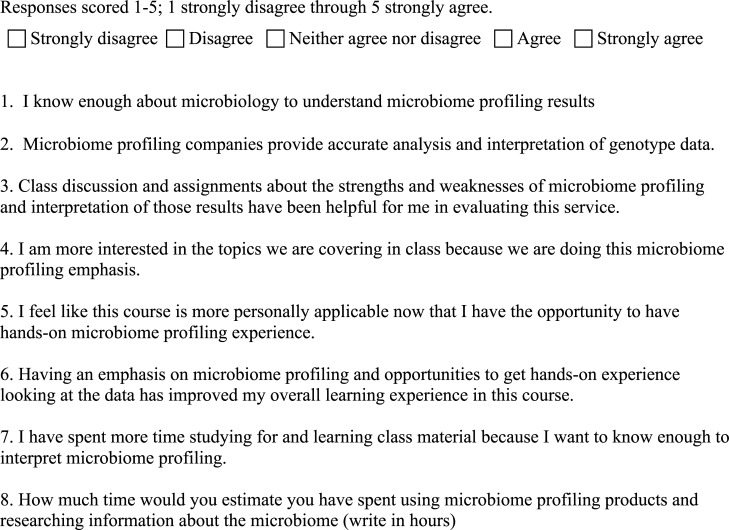
Survey questions regarding microbiome profiling experience. List of the 8 survey questions the students answered at the end of the class regarding their experience with the microbiome profiling focus in the classroom. Student attitudes were measured by agreement with statements on a 5-point Likert scale (1 = strongly disagree, 2 = disagree, 3 = Neither agree or disagree, 4 = agree, 5 = strongly agree). Results are displayed as the mean of these responses in Fig 8.

As part of this study, student attitudes towards personal microbiome testing were surveyed after our two-week focus on the microbiome (Fig 7). This survey measured their confidence in microbiome data analysis, their classroom learning experience, and their engagement with the microbiome section as well as with the course overall.

### Student confidence of microbiome data analysis

**Fig 8:**
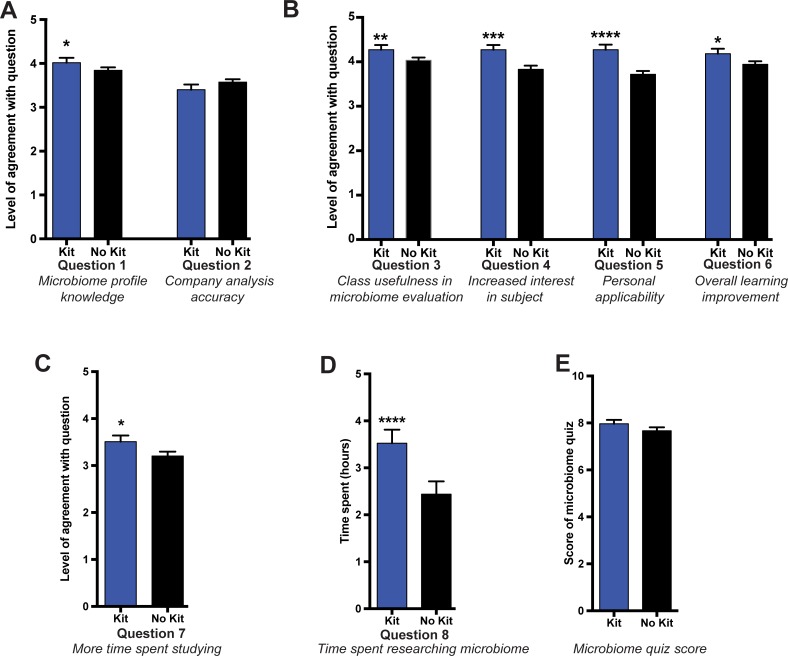
Role of personal microbiome analysis on classroom learning environment. (A) Level of agreement to question 1 and 2 about whether students “knew enough about microbiology to understand microbiome profiling results” and whether “microbiome profiling companies provide accurate analysis and interpretation of genotype data”. (B) Level of agreement to questions 3–6 about whether students felt classroom discussions had been helpful in evaluating personal microbiome data, and whether students felt having a microbiome profiling unit had resulted in greater student interest, made the course more personally applicable, and improved the overall learning experience in the course. (C) Level of agreement to question 7 about whether students had “spent more time studying for and learning class material”. (D) Level of agreement to question 8 requesting students to estimate how much time was spent using and researching microbiome profiling information and data. (E) Quiz score on the 10-question microbiome quiz. Statistical analysis performed using a Mann-Whitney U-test and all values are mean ± SEM with n = 55 for kit (blue) and n = 89 for no kit (black) (* = p<0.05; ** = p<0.01; *** = p<0.001; **** = p<0.0001).

In the post-survey students were asked to rate their level of agreement on topics regarding microbiome profiling using a 5-point Likert scale (Fig 7). Those who received a kit and evaluated their own data reported a significantly higher level of agreement that they “knew enough about microbiology to understand microbiome profiling results” compared to students evaluating demo data (p<0.05), while there was no difference in the level of agreement between groups that “microbiome profiling companies provide accurate analysis and interpretation of genotype data” (Fig 8A; questions 1 and 2).

### Microbiome profiling and classroom learning experience

Students rated their agreement towards microbiome profiling and their classroom learning experience using a 5-point Likert scale (Fig 7). Those who received a kit and evaluated their own data had significantly higher levels of agreement that “class discussion and assignments about the strengths and weaknesses of microbiome profiling and interpretation of those results has been helpful for me in evaluating this service” (p<0.01) than those evaluating demo data (Fig 8B; question 3). Those students who analyzed their own microbiome data also had significantly higher levels of agreement that they are “more interested in topics we are covering in class because we are doing this microbiome profile emphasis” than those analyzing the demo data (p<0.001) (Fig 8B; question 4). Students were also asked to rate their level of agreement regarding their attitudes towards microbiome profiling and their classroom learning experience. Students who analyzed their own microbiome data had a higher level of agreement that the course was personally applicable than those evaluating demo data (p<0.0001) (Fig 8B; question 5) and that having an emphasis on microbiome profiling had improved their overall learning in the course (p<0.05) (Fig 8B; question 6).

### Role of personal data and student engagement while researching the human microbiome

As part of survey at the end of the two-week focus on the microbiome, students were asked questions regarding their engagement in the course overall due to the two-week microbiome focus (Fig 7). To measure student engagement, students were asked in the survey to report their level of agreement that they spent more time “studying and learning class material because I want to know enough to interpret microbiome profiling,” and to estimate the time they spent using or researching personal microbiome products at the end of our two-week microbiome unit. Students analyzing their own microbiome data had significantly higher level of agreement that they had spent more time studying and learning to prepare to interpret the microbiome profiling compared to those evaluating demo data (p<0.05) (Fig 8C; question 7). Students analyzing their own microbiome data reported spending significantly more time researching the microbiome, almost 1 hour more on average, than students who did not receive a kit (p<0.0001) (Fig 8D; question 8). We also examined student familiarity with the human microbiome (i.e., measured learning, rather than self-report) in the post-surveys with a 10-question microbiome-based quiz (S3 Fig). Comparison of the scores between the groups showed no significant difference on actual learning whether or not they received a kit (Fig 8E).

## Discussion

In order to improve the effectiveness of instruction, finding relevant ways to apply active learning in the classroom has become important in all disciplines [41]. As sequencing costs have decreased and genetic tests have become more common, science and medical educators have increased the frequency of implementation of personal data analysis in the classroom [42–44]. Implementation of personal genomics analysis into the classroom has occurred at numerous schools including Stanford, Pennsylvania State University, University of Pennsylvania, Duke and others [20, 45, 46]. Analysis of genetics and race using next generation sequencing has recently been used successfully in high school classrooms to improve student learning [47]. While personal genomics is now a mature field, personal microbiome analysis is a much younger field with less known regarding data interpretation. A recent review article addressed the potential value of using the microbiome in the classroom to teach cutting edge technologies such as high throughput sequencing and bioinformatics in a personally relevant manner [48]. To our knowledge, our study is the first that has actually evaluated the benefits of incorporating personal microbiome kits into undergraduate science courses and the effect of personal microbiome data analysis on student engagement and interest. Our study is also unique from previous personal data studies in its extensive quantification of motivation both of the course overall and while students worked on an online module, providing in depth analysis of numbers of websites visited, hypotheses generated, and engagement and interest.

We previously reported that when incorporating personal genomics testing into undergraduate courses that student anticipation of analyzing their genomic data increased student interest and genetics quiz scores [25]. While personal genomics data are fixed, personal microbiome data can fluctuate based upon environmental conditions. Thus, we hypothesized that the dynamic nature of personal microbiome data would provide additional motivation to learn. Our study was designed to specifically quantify this motivation for the entire course. In addition to the end of the unit survey as was done with the personal genomics study, we also quantified motivation while students completed online learning modules analyzing their own data. Students were surveyed multiple times while completing online microbiome modules and asked to give quantifiable evidence regarding their actions and attitudes while completing the assignment and were specifically asked how engaged they were at that moment. While this study was not designed to directly compare anticipation of personal genomics analysis with classroom personal microbiome analysis on student learning, through our focus on quantifying motivation we found that incorporation of personal microbiome analysis is an effective method to improve student interest and engagement. While we did not see significantly improved microbiome quiz scores, this may be due to the fact that the quiz used to evaluate personal microbiome learning was not discriminating of the learning that actually took place. In contrast to our genetics study where students took the same pre and post genetics quiz and the group with a genome kit still scored poorly on the post quiz (~60%), both groups of students taking the microbiome quiz performed very well (~80%). Based on the motivation data, it is clear that the students analyzing their own personal microbiome data spent more time analyzing their data, but our 10-question microbiome quiz may have been too easy to quantify differences. While our study design was focused on student motivation, it would be useful to design future studies with more rigorous instrumentation to evaluate how increased student motivation and time spent on analysis of their microbiome may translate to learning gains.

In this study, we tested the hypothesis that personal microbiome data analysis enhances student interest and engagement. Student analysis of their own data increased their engagement and self-reported interest in online assignments and the course overall based on self-reported attitudes and increased time spent researching microbiome profiling. We also found that analyzing their own microbiome resulted in a better overall learning experience for students in the courses compared to students analyzing demo data. Of note, none of the student engagement measures reached an optimal level. However, analyzing personal data certainly increased engagement above baseline and this is an important finding. By providing personal microbiome data, it is possible that we shifted student motivation from extrinsic (motivated for a grade) to intrinsic (motivated by personal relevance of the subject) [49]. This is supported by higher reported time invested on microbiome tasks when microbiome kits were provided. However, it may be that extrinsic motivation was strong enough to compete with the benefits of intrinsic motivation, as evidenced by identical scores for both groups on the microbiome quiz (Fig 8E). This study was done at a selective private institution with a high-achieving and motivated student body. It is likely that the extrinsic motivation was enough to prompt performance; however, shifting to intrinsic motivation increased enjoyment without sacrificing test performance. Certainly, this is something to take into consideration when weighing the costs of incorporating personal microbiome data with the benefits of doing so. Our research only shows improvements in self-reported interest, engagement, personal applicability, learning, and time spent studying the microbiome. However, our brief microbiome assessment at the end of the study showed no actual differences in student knowledge. Certainly, an increase in engagement in the topic can lead to many other benefits beyond learning of content and should be considered as a potential motivator for including such authentic activities in classes.

While we found that student knowledge of the course-specific content (assessed by our 10-question quiz) was equal between groups, we did not assess the additional knowledge that they may have gained during their increased time on task. It is possible that students encountered additional information outside the content specific to the course assessment (e.g., sequencing processes, microbial diseases, effects on human health etc). Thus, it is possible that the increased time on task, increased websites visited, and increased evidence gathered had benefits for students beyond what we have showed in this study. Theory would suggest that increasing the time spent on a task should increase the knowledge gained [31].

In addition, our attitudinal survey did not test attitudes toward biology in general and students’ impressions of the applicability of science to their lives, in general. Question 5 in Fig 8B indicated that students felt this particular emphasis in class was more personally applicable by analyzing their own data. It is quite possible that this impression transfers to the ideology that science, in general, can apply to them. Knowing that they can gather and interpret data specific to their own bodies may indeed positively increase student attitudes toward science as an enterprise. Certainly, further work to ascertain this effect is needed.

Students receiving their own kit had more confidence that they could interpret the data from microbiome profiling, reflecting an increase in their perceived scientific reasoning ability, specifically that of interpreting data (Fig 8A, question 1). Interestingly, both treatment groups showed a high level of confidence in the accuracy and interpretations provided by the company of their microbiome data (Fig 8A, question 2). Unlike personal genomics companies, companies providing microbiome analysis are relatively new to the market and not widely advertised. Thus, student confidence in the companies interpretation of microbiome data is likely a reflection of the common misunderstandings of the nature of science that are prevalent among the general public, which often views science as an enterprise that produces facts and immutable truths [50].

This study has a large sample size and students from multiple classes; however, some restraints exist when attempting to broadly generalize these findings. All of this work was done at a single, highly selective, private institution. This highly motivated student body with a drive for grades may be one of the reasons that test scores do not significantly differ between treatments. It is possible that results would differ at less selective universities. Teachers would need to balance the feasibility and practicality of implementing personal microbiome analysis versus the potential learning benefit at their university before adding it as part of their curriculum. This might include potential student fees to cover the cost of the microbiome analysis. The transfer of these results to institutions with a more diverse student body would be valuable. In addition, it would be interesting to test the effects of personal microbiome data analysis on the attitudes and motivations of students just beginning their careers into science (i.e., in introductory biology classes) or even in students who do not intend to pursue the life sciences (i.e., non-majors). Despite these limitations, our study provides the first evidence we are aware of that student analysis of their own microbiome data enhances engagement, interest, and their perceived learning experience. Additional randomized studies examining incorporation of microbiome analysis in courses at multiple institutions can enhance our awareness of the role it has on undergraduate student learning.

It is critical to find engaging and practical means to improve science education and integration of personal microbiome data analysis represents a potentially effective means of doing this. It should certainly be taken into consideration that our effects with this treatment are modest and that overall motivation, although improved, was less than optimal when weighing the costs of incorporating personal microbiome analysis in the classroom. However, this is certainly a promising place to start thinking about engaging students personally in upper division biology courses. This topic could certainly apply to other disciplines such as having nutrition students analyze their own dietary patterns, having genetics students analyze their personal genomic information [25, 51], or having exercise physiology students design and test a personal physical fitness regimen. Just as all of these would, the incorporation of microbiome analysis into the classroom provides a means of initiating relevant discussions about current medical, ethical, and privacy issues and enables students to be better prepared to contribute to future policy discussions. While further evaluation of personal microbiome analysis in undergraduate classrooms is necessary, we believe it is an effective tool that should be thoughtfully incorporated into life science education.

## Supporting information

S1 Fig 17-question basic biology pre-survey quiz. (PDF)

S2 Fig Online microbiome module in PDF format. (PDF)

S3 Fig 10-question microbiome post-survey quiz. (PDF)

S1 Data Raw data for Figs 1–8 in excel format. (XLSX)

## References

[1] Ash C, Mueller K. Manipulating the Microbiota. Science. 2016;352(6285):530–1. doi: 10.1126/science.352.6285.530 . 27126033 10.1126/science.352.6285.530

[2] Human Microbiome Project C. A framework for human microbiome research. Nature. 2012;486(7402):215–21. doi: 10.1038/nature11209 ; PubMed Central PMCID: PMCPMC3377744. 22699610 10.1038/nature11209 PMC3377744

[3] Glasner ME. Finding enzymes in the gut metagenome. Science. 2017;355(6325):577–8. doi: 10.1126/science.aam7446 . 28183934 10.1126/science.aam7446

[4] Singer SR, Nielsen NR, Schweingruber HA. Biology education research: lessons and future directions. CBE Life Sci Educ. 2013;12(2):129–32. doi: 10.1187/cbe.13-03-0058 ; PubMed Central PMCID: PMCPMC3671636. 23737617 10.1187/cbe.13-03-0058 PMC3671636

[5] Redfield RJ. "Why do we have to learn this stuff?"—a new genetics for 21st century students. PLoS Biol. 2012;10(7):e1001356 doi: 10.1371/journal.pbio.1001356 ; PubMed Central PMCID: PMC3389015. 22802724 10.1371/journal.pbio.1001356 PMC3389015

[6] Walt DR, Kuhlik A, Epstein SK, Demmer LA, Knight M, Chelmow D, et al Lessons learned from the introduction of personalized genotyping into a medical school curriculum. Genet Med. 2011;13(1):63–6. doi: 10.1097/GIM.0b013e3181f872ac . 21057320 10.1097/GIM.0b013e3181f872ac

[7] Wood WB. Innovations in teaching undergraduate biology and why we need them. Annu Rev Cell Dev Biol. 2009;25:93–112. doi: 10.1146/annurev.cellbio.24.110707.175306 . 19575638 10.1146/annurev.cellbio.24.110707.175306

[8] Sender R, Fuchs S, Milo R. Are We Really Vastly Outnumbered? Revisiting the Ratio of Bacterial to Host Cells in Humans. Cell. 2016;164(3):337–40. doi: 10.1016/j.cell.2016.01.013 . 26824647 10.1016/j.cell.2016.01.013

[9] Gensollen T, Iyer SS, Kasper DL, Blumberg RS. How colonization by microbiota in early life shapes the immune system. Science. 2016;352(6285):539–44. doi: 10.1126/science.aad9378 ; PubMed Central PMCID: PMCPMC5050524. 27126036 10.1126/science.aad9378 PMC5050524

[10] Buck MD, Sowell RT, Kaech SM, Pearce EL. Metabolic Instruction of Immunity. Cell. 2017;169(4):570–86. doi: 10.1016/j.cell.2017.04.004 . 28475890 10.1016/j.cell.2017.04.004 PMC5648021

[11] Cho I, Blaser MJ. The human microbiome: at the interface of health and disease. Nat Rev Genet. 2012;13(4):260–70. doi: 10.1038/nrg3182 ; PubMed Central PMCID: PMCPMC3418802. 22411464 10.1038/nrg3182 PMC3418802

[12] Human Microbiome Project C. Structure, function and diversity of the healthy human microbiome. Nature. 2012;486(7402):207–14. doi: 10.1038/nature11234 ; PubMed Central PMCID: PMCPMC3564958. 22699609 10.1038/nature11234 PMC3564958

[13] Goodrich JK, Davenport ER, Waters JL, Clark AG, Ley RE. Cross-species comparisons of host genetic associations with the microbiome. Science. 2016;352(6285):532–5. doi: 10.1126/science.aad9379 ; PubMed Central PMCID: PMCPMC5116907. 27126034 10.1126/science.aad9379 PMC5116907

[14] Lynch SV, Pedersen O. The Human Intestinal Microbiome in Health and Disease. N Engl J Med. 2016;375(24):2369–79. doi: 10.1056/NEJMra1600266 . 27974040 10.1056/NEJMra1600266

[15] Pamer EG. Resurrecting the intestinal microbiota to combat antibiotic-resistant pathogens. Science. 2016;352(6285):535–8. doi: 10.1126/science.aad9382 ; PubMed Central PMCID: PMCPMC4984266. 27126035 10.1126/science.aad9382 PMC4984266

[16] Blaser MJ. Antibiotic use and its consequences for the normal microbiome. Science. 2016;352(6285):544–5. doi: 10.1126/science.aad9358 ; PubMed Central PMCID: PMCPMC4939477. 27126037 10.1126/science.aad9358 PMC4939477

[17] Subramanian S, Huq S, Yatsunenko T, Haque R, Mahfuz M, Alam MA, et al Persistent gut microbiota immaturity in malnourished Bangladeshi children. Nature. 2014;510(7505):417–21. doi: 10.1038/nature13421 ; PubMed Central PMCID: PMCPMC4189846. 24896187 10.1038/nature13421 PMC4189846

[18] Rhodes R. Ethical issues in microbiome research and medicine. BMC Med. 2016;14(1):156 doi: 10.1186/s12916-016-0702-7 ; PubMed Central PMCID: PMCPMC5059983. 27729053 10.1186/s12916-016-0702-7 PMC5059983

[19] Turnbaugh PJ, Ley RE, Hamady M, Fraser-Liggett CM, Knight R, Gordon JI. The human microbiome project. Nature. 2007;449(7164):804–10. doi: 10.1038/nature06244 ; PubMed Central PMCID: PMCPMC3709439. 17943116 10.1038/nature06244 PMC3709439

[20] Zhang TR, Anderson MA. Personalized genetic testing as a tool for integrating ethics instruction into biology courses. J Microbiol Biol Educ. 2014;15(2):197–201. doi: 10.1128/jmbe.v15i2.773 ; PubMed Central PMCID: PMC4278477. 25574278 10.1128/jmbe.v15i2.773 PMC4278477

[21] Salari K, Pizzo PA, Prober CG. Commentary: to genotype or not to genotype? Addressing the debate through the development of a genomics and personalized medicine curriculum. Acad Med. 2011;86(8):925–7. doi: 10.1097/ACM.0b013e3182223acf . 21795901 10.1097/ACM.0b013e3182223acf

[22] Boguski MS, Boguski RM, Berman MR. Personal genotypes are teachable moments. Genome Med. 2013;5(3):22 doi: 10.1186/gm426 ; PubMed Central PMCID: PMC3706876. 23514125 10.1186/gm426 PMC3706876

[23] Callaway E. Microbiome privacy risk. Nature. 2015;521(7551):136 doi: 10.1038/521136a . 25971486 10.1038/521136a

[24] Salari K, Karczewski KJ, Hudgins L, Ormond KE. Evidence that personal genome testing enhances student learning in a course on genomics and personalized medicine. PLoS One. 2013;8(7):e68853 doi: 10.1371/journal.pone.0068853 ; PubMed Central PMCID: PMC3720862. 23935898 10.1371/journal.pone.0068853 PMC3720862

[25] Weber KS, Jensen JL, Johnson SM. Anticipation of Personal Genomics Data Enhances Interest and Learning Environment in Genomics and Molecular Biology Undergraduate Courses. PLoS One. 2015;10(8):e0133486 doi: 10.1371/journal.pone.0133486 ; PubMed Central PMCID: PMCPMC4524698. 26241308 10.1371/journal.pone.0133486 PMC4524698

[26] Schiefele U. Interest and learning from text. Sci Stud Read. 1999;3:257–80.

[27] Williams KC, Williams CC. Five key ingredients for improving student motivation. Research in Higher Education. 2011;12:1–23.

[28] Frey N, Fisher D. Motivation Requires a Meaningful Task. English Journal. 2010;100(1):30–6.

[29] HSaR AK. The Four-Phase Model of Interest Development. Educational Psychologist 2006;41(2):111–27.

[30] Eccles J AT, Futterman R, Goff SB, Kaczala CM, Meece JL, and, C. M. Expectancies, values, and academic behaviors In Spence JT (Ed), Achievement and Achievement Motives San Francisco: W. H. Freeman; 1983 p. 75–146.

[31] Bransford JD BA, and Cocking RR. How people learn: brain, mind, experience, and school Washington, D.C.: National Academy Press; 2000. 206–30 p.

[32] RRaD EL. Self-determination theory and the facilitation of intrinsic motivation, social development, and well-being. American Psychologist. 2000;(55):68–78. 11392867 10.1037//0003-066x.55.1.68

[33] DEaR RM. The “what” and “why” of goal pursuits: Human needs and the self-determination of behavior. Psychological Inquiry. 2000;11:227–68.

[34] DEaR RM. Intrinsic motivation and self-determination in human behavior New York, NY:: Plenum; 1985.

[35] Ryan RM CJ, and Plant RW. Emotions in nondirected text learning. Learning and Individual Differences. 1990;2(1):1–17.

[36] O JE. Educational psychology: Developing learners Upper Saddle River, NJ: Pearson Merrill Prentice Hall; 2006.

[37] Priniski SJ HCaHJ. Making Learning Personally Meaningful: A New Framework for Relevance Research. The Journal of Experimental Education. 2018;86(1):11–29. 10.1080/00220973.2017.1380589 PMC6191053 30344338

[38] Houtman JHS. Breakthroughs in Bioscience. The Human Microbiome: Your Own Personal Ecosystem. The Federation of American Societies for Experimental Biology. 2015:1–13.

[39] Hansen TH, Gobel RJ, Hansen T, Pedersen O. The gut microbiome in cardio-metabolic health. Genome Med. 2015;7(1):33 doi: 10.1186/s13073-015-0157-z ; PubMed Central PMCID: PMCPMC4378584. 25825594 10.1186/s13073-015-0157-z PMC4378584

[40] Shi J, Wood WB, Martin JM, Guild NA, Vicens Q, Knight JK. A diagnostic assessment for introductory molecular and cell biology. CBE Life Sci Educ. 2010;9(4):453–61. doi: 10.1187/cbe.10-04-0055 ; PubMed Central PMCID: PMCPMC2995763. 21123692 10.1187/cbe.10-04-0055 PMC2995763

[41] Freeman S, Eddy SL, McDonough M, Smith MK, Okoroafor N, Jordt H, et al Active learning increases student performance in science, engineering, and mathematics. Proc Natl Acad Sci U S A. 2014;111(23):8410–5. Epub 2014/05/14. doi: 10.1073/pnas.1319030111 ; PubMed Central PMCID: PMCPMC4060654. 24821756 10.1073/pnas.1319030111 PMC4060654

[42] Hagiwara N. Application of active learning modalities to achieve medical genetics competencies and their learning outcome assessments. Adv Med Educ Pract. 2017;8:817–29. Epub 2017/12/26. doi: 10.2147/AMEP.S145696 ; PubMed Central PMCID: PMCPMC5733911. 29276425 10.2147/AMEP.S145696 PMC5733911

[43] Plunkett-Rondeau J, Hyland K, Dasgupta S. Training future physicians in the era of genomic medicine: trends in undergraduate medical genetics education. Genet Med. 2015;17(11):927–34. Epub 2015/02/13. doi: 10.1038/gim.2014.208 . 25674779 10.1038/gim.2014.208

[44] Katsanis SH, Dungan JR, Gilliss CL, Ginsburg GA. Educating future providers of personalized medicine. N C Med J. 2013;74(6):491–2. Epub 2013/12/10. . 24316773

[45] Garber KB, Hyland KM, Dasgupta S. Participatory Genomic Testing as an Educational Experience. Trends Genet. 2016;32(6):317–20. Epub 2016/04/28. doi: 10.1016/j.tig.2016.03.008 . 27117243 10.1016/j.tig.2016.03.008

[46] Weitzel KW, McDonough CW, Elsey AR, Burkley B, Cavallari LH, Johnson JA. Effects of Using Personal Genotype Data on Student Learning and Attitudes in a Pharmacogenomics Course. Am J Pharm Educ. 2016;80(7):122 Epub 2016/10/21. doi: 10.5688/ajpe807122 ; PubMed Central PMCID: PMCPMC5066925. 27756930 10.5688/ajpe807122 PMC5066925

[47] Yang X, Hartman MR, Harrington KT, Etson CM, Fierman MB, Slonim DK, et al Using Next-Generation Sequencing to Explore Genetics and Race in the High School Classroom. CBE Life Sci Educ. 2017;16(2). Epub 2017/04/15. doi: 10.1187/cbe.16-09-0281 ; PubMed Central PMCID: PMCPMC5459240. 28408407 10.1187/cbe.16-09-0281 PMC5459240

[48] Hartman MR, Harrington KT, Etson CM, Fierman MB, Slonim DK, Walt DR. Personal microbiomes and next-generation sequencing for laboratory-based education. FEMS Microbiol Lett. 2016;363(23). Epub 2016/11/20. doi: 10.1093/femsle/fnw266 . 27856569 10.1093/femsle/fnw266 PMC5827621

[49] Eccles JS, Wigfield A. Motivational beliefs, values, and goals. Annual Review of Psychology. 2002;53:109–32. doi: 10.1146/annurev.psych.53.100901.135153 11752481 10.1146/annurev.psych.53.100901.135153

[50] Parker L, Krockover GH, Lasher-Trapp S, & Eichinger DC. Ideas About the Nature of Science Held by Undergraduate Atmospheric Science Students. American Meteorological Society. 2008:1681–8. doi: 10.1175/2008BAMS2349.1

[51] Jensen JL BE, Kummer TA, and Weber KS. Using Backward Design in Education Research: A Research Methods Essay. Journal of Microbiology and Biology Education. 2017;18(3). https://doi.org/10.1128/jmbe.v18i3.1367. 10.1128/jmbe.v18i3.1367 PMC5976040 29854045

