# Supplementary material for: Personal microbiome analysis improves student engagement and interest in Immunology, Molecular Biology, and Genomics undergraduate courses
Source: PLoS One. 2018 Apr 11;13(4):e0193696. doi: 10.1371/journal.pone.0193696 (PMC5894996; doi:10.1371/journal.pone.0193696)
Supplement: S1 Fig — (PDF) [file pone.0193696.s001.pdf]

Supplemental Figure 1

1. Many infectious diseases are becoming difficult to treat because of bacterial resistance to antibiotics. Populations of bacteria can become resistant when they are exposed to an antibiotic. What is the best general explanation for how this occurs?
    a. The antibiotic induces specific mutations in some of the bacteria that make them antibiotic-resistant.
    b. The antibiotic activates bacterial genes encoding enzymes that can destroy the antibiotic.
    c. The antibiotic increases the bacterial mutation rate, so that resistant mutant bacteria are more likely to arise.
    d. Antibiotic-resistant mutant bacteria already present in the population survive and reproduce in the presence of the antibiotic.

2. Which of the following statements comparing bacteria and eukaryotes is true?
    a. Eukaryotic cells have a nucleus surrounded by a nuclear membrane; bacterial cells don't.
    b. Eukaryotic cells don't have cell walls; many bacterial cells do.
    c. The genetic material of eukaryotic cells is DNA; the genetic material of bacteria can be either RNA or DNA.
    d. Eukaryotic cells use a different code to specify the amino acids in proteins than bacterial cells.

3. Which of the following statements about viruses is <u>FALSE</u>?
    a. Viruses have a nucleus but no cytoplasm.
    b. Viruses can reproduce only when they are inside a living host cell.
    c. Viruses cannot make proteins on their own.
    d. Some viruses use RNA rather than DNA as their genetic material.

4. Consider a short polar charged region and a short non-polar region in a long polypeptide chain. When dissolved in water, the polypeptide will most likely fold to form a protein in which:
    a. The non-polar region is exposed on its surface and the polar region is interior.
    b. The polar region is exposed on its surface and the non-polar region is interior.
    c. Both the non-polar and the polar region are exposed on its surface.
    d. Both the non-polar region and the polar region are interior.

5. If green algae cells in a buffer solution containing only inorganic salts are placed in a sealed container at room temperature with excess carbon dioxide gas and exposed to light, the cells will
    a. Live for many hours and multiply.
    b. Live for several hours, but fail to multiply because there is no source of carbon in the buffer solution.
    c. Live for several hours, but fail to multiply because no oxygen is present.
    d. Die rapidly, because no oxygen is present.

The molecular structures shown below are representative of five major classes of building-block molecules (monomers) that make up macromolecules and membranes in cells. Match each structure with the name of the correct monomer (a – e).

a) fatty acid   b) amino acid   c) nucleotide   d)phospholipid   e)monosaccharide

6. 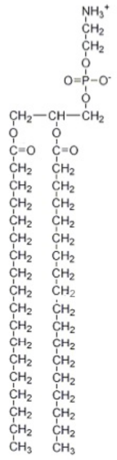

7. 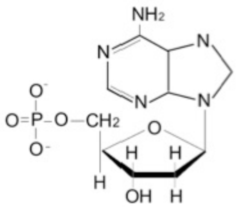

8. 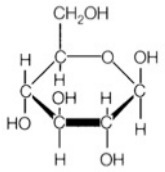

9. 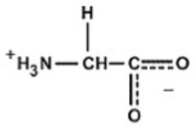

10. 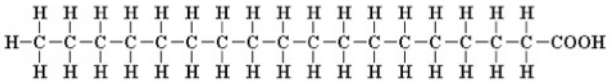

11. Consider the following chemical reaction: A + B ⇔ C. In the diagram below, the dashed line represents the energetics of this reaction WITHOUT an enzyme. Which of the solid lines (a, b, c) in the diagram best represents the way the curve would look in the presence of an enzyme catalyst that increases the reaction rate?

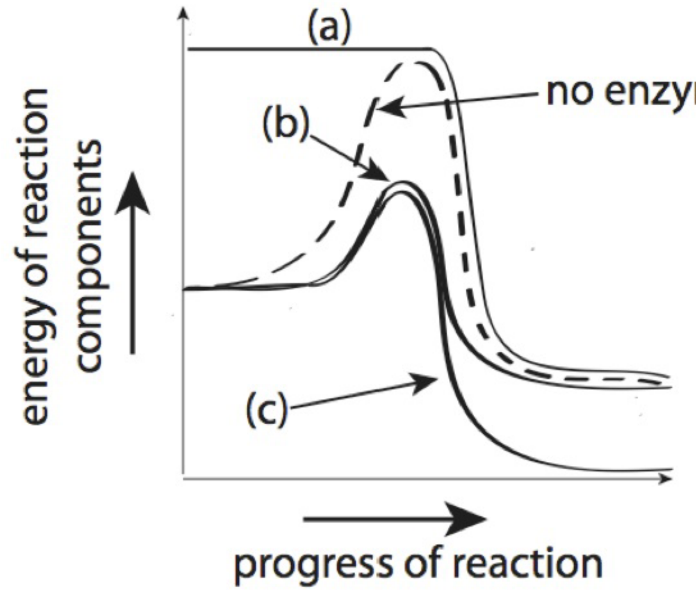

12. Which of the following substances will be least likely to diffuse through a pure phospholipid bilayer membrane that contains no proteins?

a) Na^+

b) 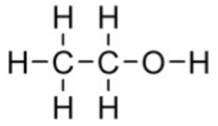

c) 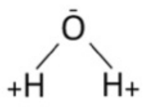

d) O=O

13. The photograph below shows a single replicated chromosome (consisting of two sister chromatids) just before mitosis. This chromosome contains:
    a. Two single-stranded DNA molecules.
    b. One double-stranded DNA molecule.
    c. Two double-stranded DNA molecules.
    d. Many double-stranded DNA molecules.

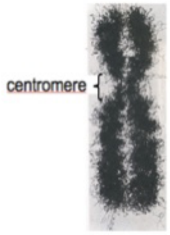

14. The replicated chromosome shown above contains:
    a. DNA from one of your parents in the sister chromatid on the left and DNA from the other parent in the sister chromatid on the right.

b. DNA contributions from both parents, resulting from recombination (crossing over).
c. DNA from only one of your parents.

15. Which of the following statements about DNA synthesis at the replication fork of a replicating DNA molecule is FALSE?
    a. Nucleotides are added at the 3' ends of all the new strands in a replicating DNA molecule.
    b. Double-stranded DNA synthesis requires both deoxyribonucleotides and ribonucleotides.
    c. The sequence of each newly synthesized single strand is identical to that of the parental single strand that served as its template.
    d. One of the two new strands must be synthesized in fragments because the two strands have opposite directionality.

16. The human hexokinase enzyme has the same function as the bacterial hexokinase enzyme but is somewhat different in its amino acid sequence. You have obtained a mutant bacterial strain in which the gene for hexokinase and its promoter are missing. If you introduce into your mutant strain a DNA plasmid engineered to contain the coding sequence of the human hexokinase gene, driven by the normal bacterial promoter, the resulting bacteria will now produce:
    a. The bacterial from of hexokinase.
    b. The human form of hexokinase.
    c. A hybrid enzyme that is partly human, partly bacterial.
    d. Both forms of the enzyme.

17. In a certain mutant strain of bacteria, the enzyme leucyl-tRNA synthetase mistakenly attaches isoleucine to leucyl-tRNA 10% of the time instead of attaching leucine. These bacteria will synthesize:
    a. Proteins in which leucine is inserted at some positions normally occupied by isoleucine.
    b. Proteins in which isoleucine is inserted at some positions normally occupied by leucine.
    c. No abnormal proteins, because the ribosomal translation machinery will recognize the inappropriately activated tNRAs and exclude them from the translation process.
    d. No proteins, because the inappropriately activated tRNAs will block translation.
