# Supplementary material for: Personal microbiome analysis improves student engagement and interest in Immunology, Molecular Biology, and Genomics undergraduate courses
Source: PLoS One. 2018 Apr 11;13(4):e0193696. doi: 10.1371/journal.pone.0193696 (PMC5894996; doi:10.1371/journal.pone.0193696)
Supplement: S2 Fig — (PDF) [file pone.0193696.s002.pdf]

Supplemental Figure 2

Assignment #1

In this project you will access data from the uBiome website and learn about the microboime and microbial diversity in the gut and other body parts.

Directions:

1. Log onto the uBiome website (ubiome.com) using your login information.

[If you do not have your own data then login using the following demo info (email: preview@ubiome.com password: ilovebacteria). The **demo** data is for a 40-year-old male omnivore who has 2 kids, a dog, drinks alcohol once a month, takes antibiotics infrequently, and exercises regularly (this information will be important later).]

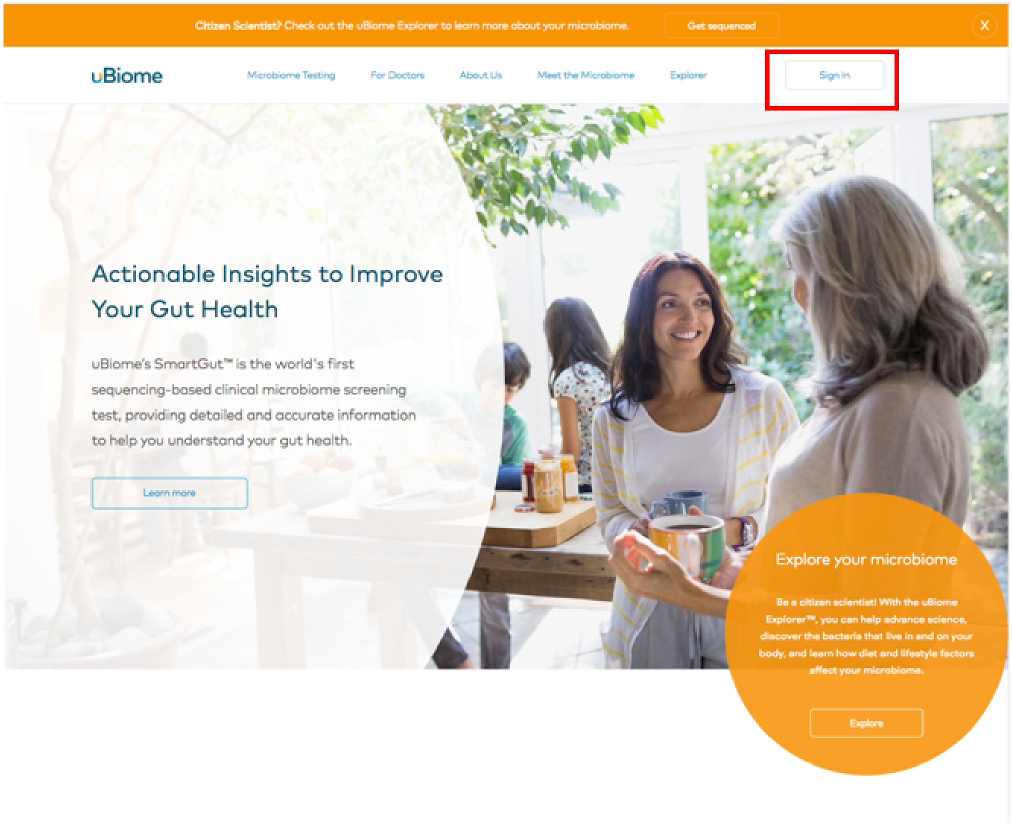

2. Once logged in, choose "Explore Your Data".

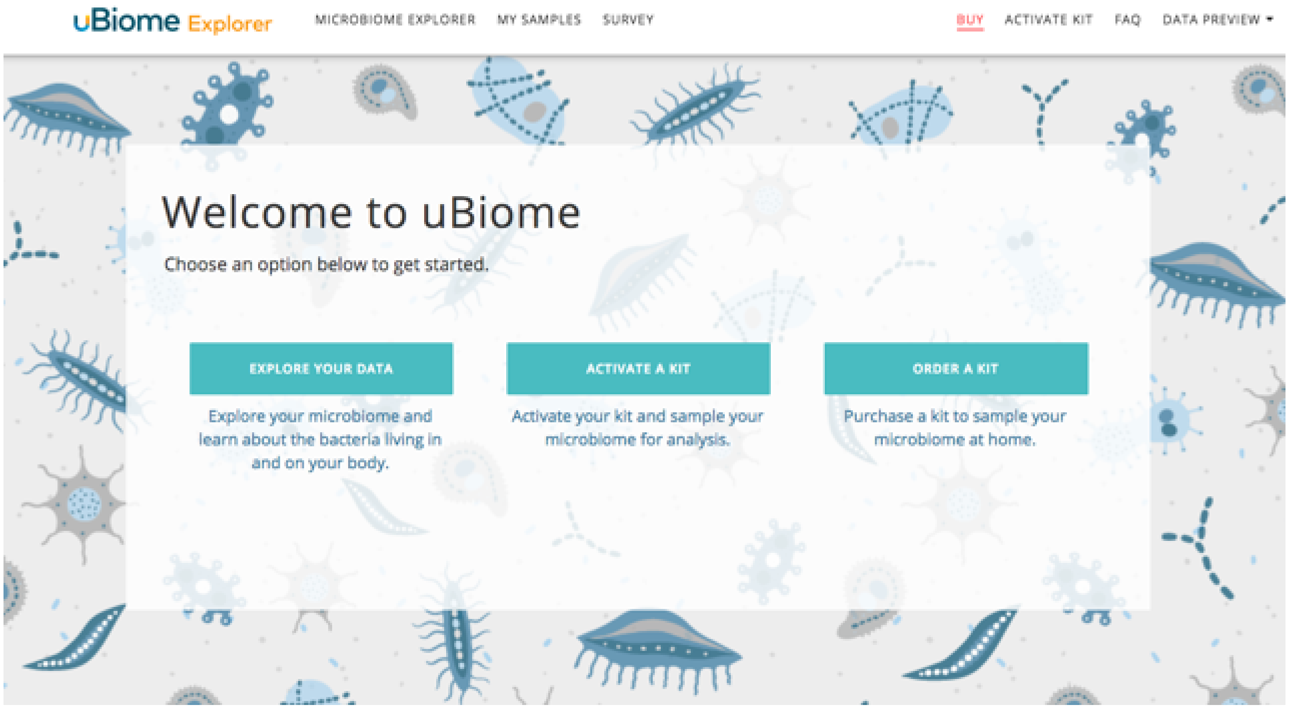

The default sample type is "The Gut". Keep this. These questions refer to the Gut. At the top of the page, click on "My Bacteria".

What is the most abundant bacterial phylum in the gut sample you are viewing?

What percentage of the total bacteria is the most abundant bacterial phylum?

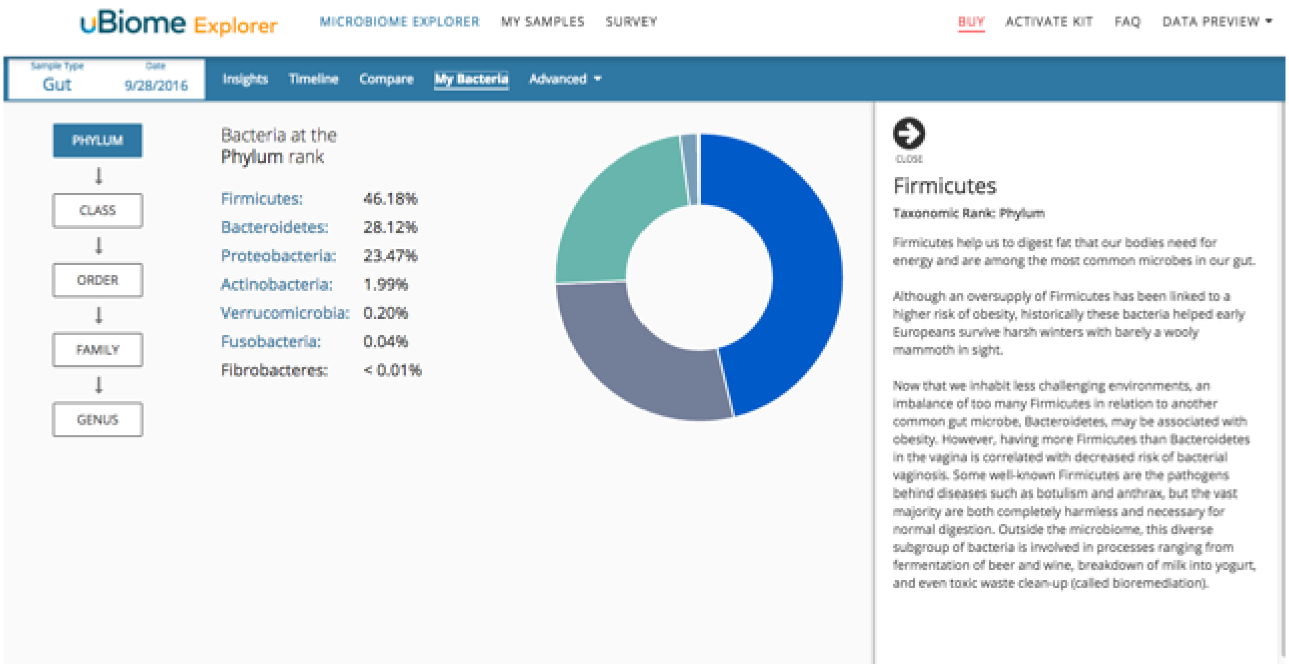

On the right-hand side is a description of the most abundant bacterial phylum.

What is the hypothesized function of this phylum of bacteria in the gut?

Perform a Google search and find at least one piece of evidence that suggests this bacterial phylum influences health. Write down your evidence (and please include the website address of the source).

Referring to the Google search you just performed:

On a scale of 0 to 5 (0 = not at all, 5 = extremely),

	0	1	2	3	4	5
how engaged were you in searching for these health influences (e.g., how excited were you to do the search, how interested were you in the results)?						

Approximately how much time did you spend searching on Google?

More than 1 hour

30 -45 minutes

15 - 30 minutes

5 - 15 minutes

Less than 5 minutes

Approximately how many websites did you visit looking for information?

More than 10

6 - 10

2 - 5

Only 1

You were only asked to record one piece of evidence in the above question. How much evidence did you actually find in your searching?

I found more than 5 sources of evidence

I found 3 - 5 sources of evidence

I found 2 - 3 sources of evidence

I found only the one piece that I needed to answer the question

Assignment #2

Click on the "Compare" tab at the top of the page. It will bring you to this page:

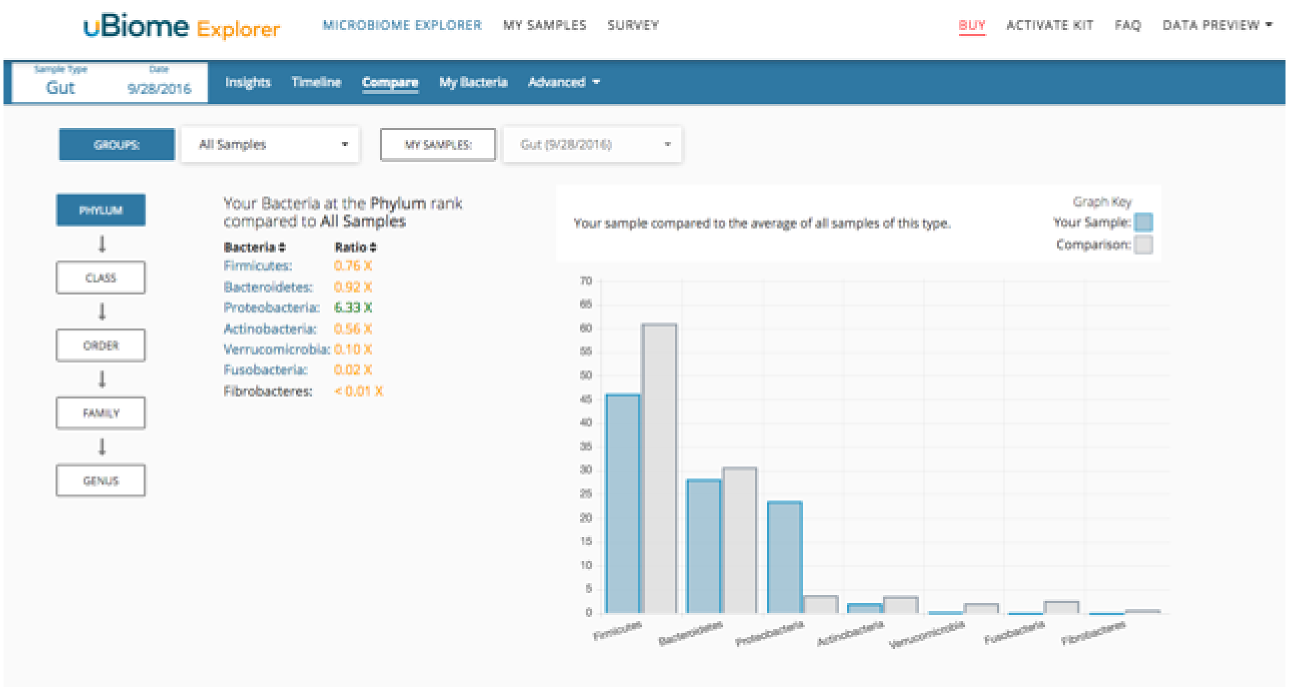

Click on the "All Samples" drop-down menu and compare the sample gut profile you are viewing to the website sub groups profiles (e.g. Vegetarians, Paleo Diet, Omnivores, etc.). Which sub group does your sample's gut profile most resemble?

Does this make sense based on the diet or other factors of you (or of the sample person you are viewing*)? Please explain.

* remember, the **demo** data is for a 40-year-old male omnivore who has 2 kids, a dog, drinks alcohol once a month, takes antibiotics infrequently, and exercises regularly

Using Google again, find evidence to support your answer to the previous question (i.e., how the profile matches or doesn't match diet or other factors). Please list at least one piece of evidence below and include the website address).

Referring to the Google search you just performed:

On a scale of 0 to 5 (0 = not at all, 5 = extremely),

	0	1	2	3	4	5
how engaged were you in searching for the relationship between gut microbes and diet (e.g., how excited were you to do the search, how interested were you in the results)?						

Approximately how much time did you spend searching on Google?

More than 1 hour

30 -45 minutes

15 - 30 minutes

5 - 15 minutes

Less than 5 minutes

Approximately how many websites did you visit looking for information?

More than 10

6 - 10

2 - 5

Only 1

You were only asked to give one piece of evidence. How much evidence did you actually find in your searching?

I found more than 5 sources of evidence

I found 3 - 5 sources of evidence

I found 2 - 3 sources of evidence

I found only the one piece that I needed to answer the question

Click on the "Insights" tab and then select the 'Go to Diversity' box on the left-hand-side of the page to go to the Microbiome diversity sections as shown below:

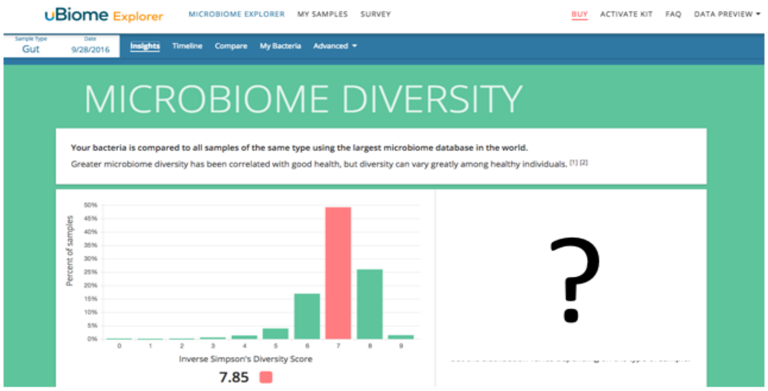

What percentage of gut samples in their database are less diverse than your sample?

Click on the "Compare" tab once again and again choose "Genus" for the level of comparison. Look at the relative diversity in *your* sample compared to 'Heavy Drinkers'. (Remember, to get to the different diet types, click on "All Samples".) Which genus is *most decreased* in your sample in comparison to 'Heavy Drinkers'? (Hint: For this question, do not refer to the bar graph since it only shows the most abundant genera; instead, refer to the list to the left of the bar graph)

Come up with a hypothesis to explain why this genus would be less in your sample than in a 'Heavy Drinker's" sample.*

* Again, remember that if you are using **demo** data, it is for a 40-year-old male omnivore who has 2 kids, a dog, drinks alcohol once a month, takes antibiotics infrequently, and exercises regularly

Assignment #3

The uBiome website gives information on different metabolic processes based on the gut profile you are viewing (example below). To get to this page, click on the "Advanced" tab at the top and choose "Function".

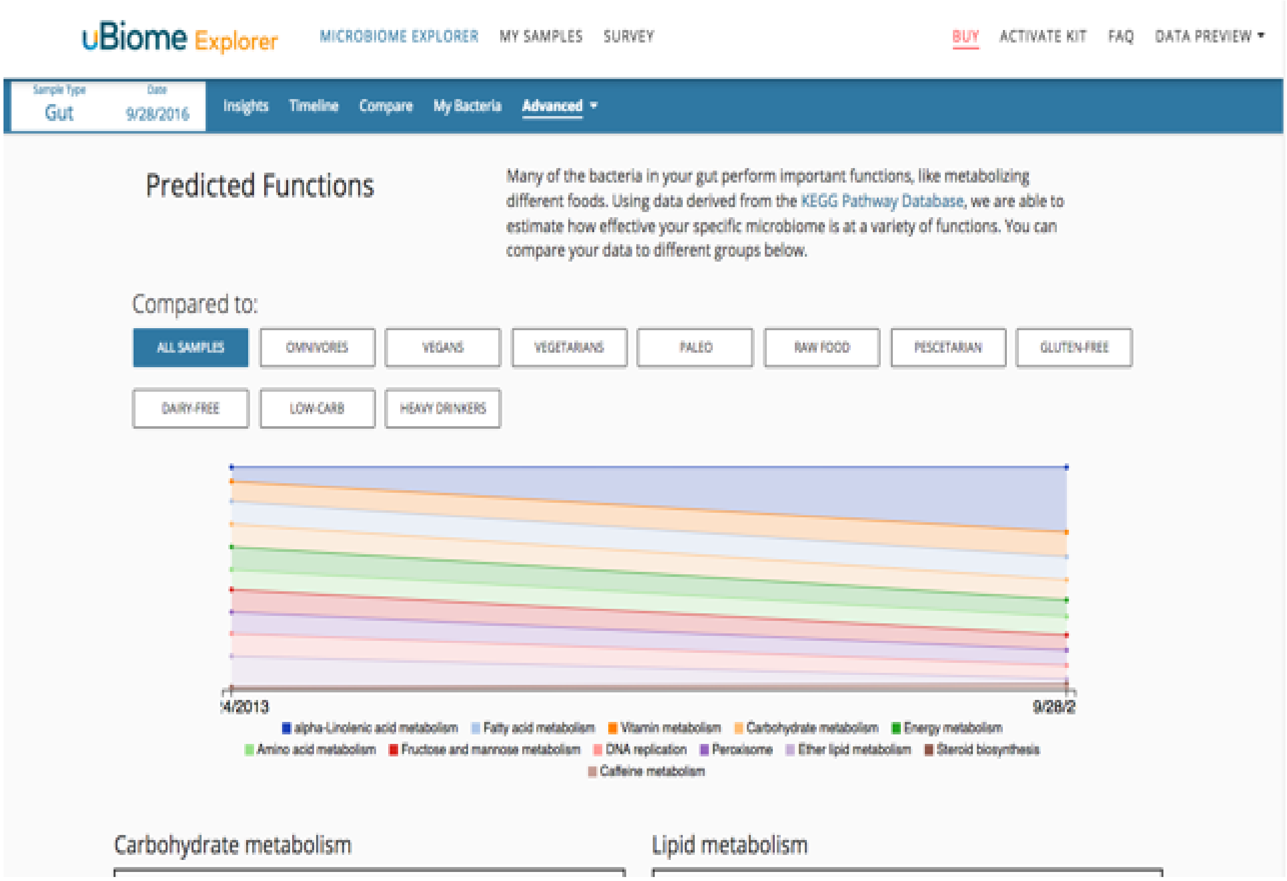

Is this information reliable or not? Justify your answer with support from a Google search. (Please include website address(es).)

Referring to the Google search you just performed:

On a scale of 0 to 5 (0 = not at all, 5 = extremely),

	0	1	2	3	4	5
how engaged were you in finding out whether or not these results are reliable (e.g., how excited were you to do the search, how interested were you in the results)?						

Approximately how much time did you spend searching on Google?

More than 1 hour

30 -45 minutes

15 - 30 minutes

5 - 15 minutes

Less than 5 minutes

Approximately how many websites did you visit looking for information?

More than 10

6 - 10

2 - 5

Only 1

In answering this reliability question, how many sources did you consult?

I found more than 5 sources

I found 3 - 5 sources

I found 2 - 3 sources

I found only the one source that I needed to answer the question

Assignment #4

Click on "Advanced" again and choose "Tree". It should look as follows:

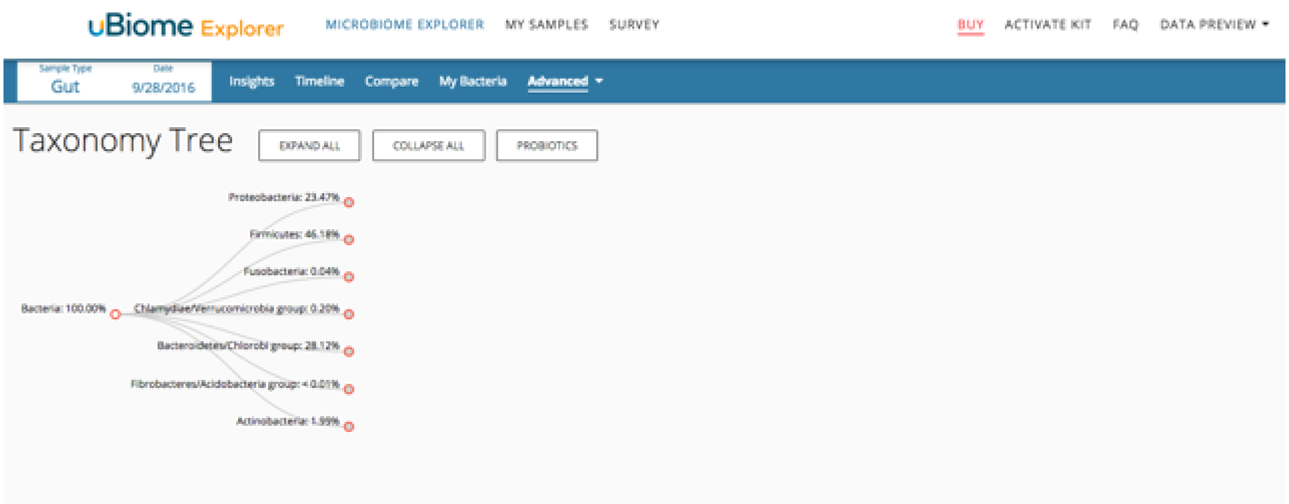

Hovering your mouse over any of the circles will expand and show the bacterial components of that group.

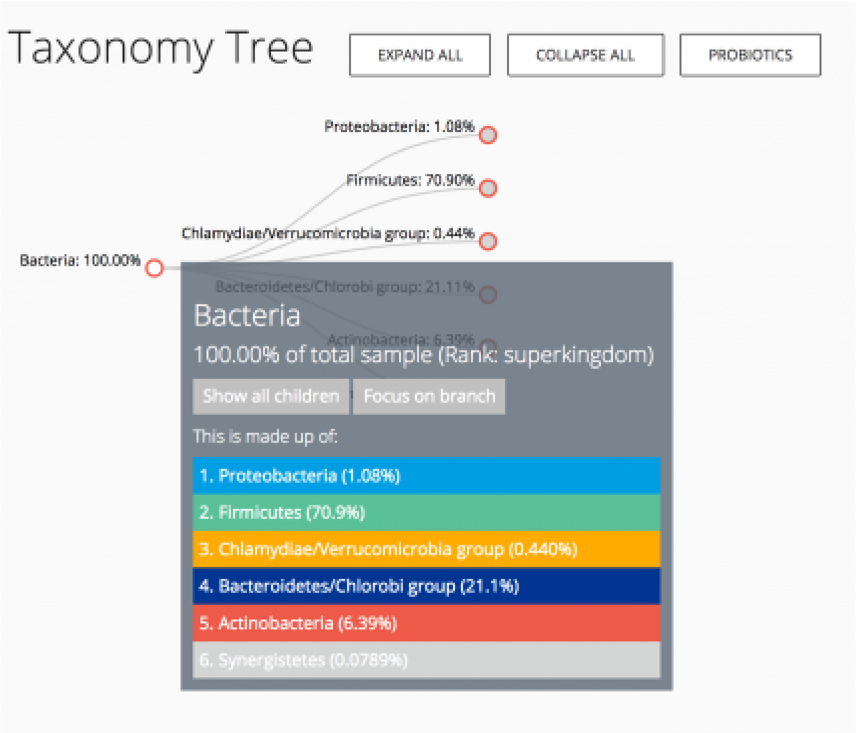

Choose the most abundant bacterial phylum and click on that phylum's circle. Then choose the most abundant bacterial class and do the same. Keep moving down the phylogenetic classifications choosing the most abundant until you can go no further.

For the most abundant gut phylum in the sample you are viewing, what is the most abundant type of bacteria when you look at the lowest phylogenetic classification (i.e., genus, species, subspecies …)?

Offer one hypothesis about a lifestyle, medical condition or dietary habit that would explain these results (you may have to Google again).

Referring to the hypothesis you just gave for the relationship between the presence of that microbial species in the sample and lifestyle, medical, or dietary habits:

On a scale of 0 to 5 (0 = not at all, 5 = extremely),

	0	1	2	3	4	5
how engaged were you in searching for this (e.g., how excited were you to do the search, how interested were you in the results)?						

Approximately how much time did you spend searching on Google/coming up with your hypothesis?

More than 1 hour

30 -45 minutes

15 - 30 minutes

5 - 15 minutes

Less than 5 minutes

Approximately how many websites did you visit looking for information?

More than 10

6 - 10

2 - 5

Only 1

I did not look online

You were only asked to offer just one hypothesis. How many hypotheses did you actually come up with in your searching?

I came up with more than 5 hypotheses

I came up with 3 - 5 hypotheses

I came up with 2 - 3 hypotheses

I only came up with one hypothesis that I needed to answer the question

Evaluation of entire assignment

Referring to the **entire assignment** that you just completed,

Scale: 0 = Not at all; 5 = Extremely

	0	1	2	3	4	5
how engaging was it (i.e., how much did it capture your interest; how interested were you in learning the information)?						
how much did you enjoy doing the assignment?						

how interested are you in learning more information about this topic?

how likely are you to learn more about this topic on your own time?

Referring to the **entire assignment** that you just completed, how much total time did it take you to complete (including any web searches you performed)?

More than 3 hours

2 - 3 hours

1 - 2 hours

30 minutes - 1 hour

Less than 30 minutes

For what course did you complete this assignment?

MMBIO 442

MMBIO 463

MMBIO 468

Did you use your own gut data or the 'Demo data' for this microbiome module?

I used my own microbiome data.

I used the Demo data because my gut data is not available yet.

I used the Demo data because I did not have my microbiome sequenced.

In order to receive credit for this assignment, please type your name below:
