# Supplementary material for: Personal microbiome analysis improves student engagement and interest in Immunology, Molecular Biology, and Genomics undergraduate courses
Source: PLoS One. 2018 Apr 11;13(4):e0193696. doi: 10.1371/journal.pone.0193696 (PMC5894996; doi:10.1371/journal.pone.0193696)
Supplement: S3 Fig — (PDF) [file pone.0193696.s003.pdf]

Supplemental Figure 3

1. Metabolites that are produced by microbes in the gut can affect host health by…
    a. Leaking through the gut mucosal barrier and into the blood stream
    b. Increasing systemic inflammation
    c. Promoting atherosclerosis
    d. All of the above
    e. None of the above

2. Which of the following cannot be revealed by 16S rRNA-based gut microbiota profiling?
    a. Which taxa (phylotypes) are present
    b. The rate of proliferation of the taxa present
    c. The relative abundance of the taxa present
    d. The phylogenetic relationship between the taxa present

3. Mouse and/or human studies indicate that a state of bacterial dysbiosis in the gut can contribute to which of the following? (mark all that apply)
    a. Type 2 diabetes
    b. Athlete's foot
    c. Obesity
    d. Colorectal cancer
    e. Depression
    f. The efficacy of a melanoma cancer treatment
    g. Autism behaviors
    h. Anxiety
    i. Breast cancer
    j. Physiological response to stress

4. How can prebiotics shape the gut microbiota if they cannot be digested by humans?
    a. Some gut bacteria can digest and metabolize prebiotics, and those that can will proliferate and thus shift the composition of the gut microbiota
    b. Prebiotics are usually contaminated with bacterial species that colonize the gut and displace other kinds of bacteria.
    c. Prebiotics cannot shape the gut microbiota—any perceived benefit is due to placebo effects
    d. Prebiotics kill pathogenic bacteria, allowing beneficial bacteria to take its place

5. Which two phyla are major components of the human gut microbiota?
    a. Methanobacteria and Actinobacteria
    b. Proteobacteria and Streptococcus
    c. Firmicutes and Bacteriodetes
    d. Staphylococcus and Arabidopsis

6. How does changing the diet change the composition of the gut microbiota?
    a. New foods introduce new kinds of bacteria into the gut
    b. New foods can be poisonous to certain kinds of bacteria
    c. Bacteria that have the genetic capability to utilize nutrients from the new foods will proliferate, while those that don't will become less abundant
    d. The composition of the gut microbiota becomes fixed by 2-3 years of age and cannot be changed by diet

7. Studies have shown that “germ-free mice need to consume approximately 30% more calories to sustain a body weight similar to conventionally raised mice.” Given what we know about gut microbes, which of the following is the most likely explanation for this phenomenon?
    a. Normal mice obtain calories by digesting the bacteria in their gut, whereas germ-free mice cannot
    b. Germ-free mice have a genetically higher metabolic rate than conventional mice and therefore must consume more calories
    c. Germ-free mice are harvesting less of the energy from their food because they cannot digest as much of it
    d. Germ-free mice cannot digest any food until it reaches the large intestine, which reduces the time available for harvesting calories from the food.

8. If a 12-year-old boy from rural Brazil who was raised primarily on a plant-based diet were adopted and brought to New York City where he adopted a western-style diet, what would likely happen to the diversity of the boy’s gut microbiota?
    a. Diversity will likely decrease in response to a high-protein/high-fat western-style diet
    b. Diversity will likely increase in response to a high-protein/high-fat western-style diet
    c. Diversity will likely remain unchanged since it is fixed by 2-3 years of age and remains constant from then on
    d. The direction of his change in microbial diversity will depend on his genetic background

9. Administration of antimicrobial agents, including broad-spectrum antibiotics, has been proposed as a possible contributor to the obesity epidemic in the western world. Why?
    a. Because antibiotics introduce new, more diverse, opportunistic gut microbes that can lead to obesity
    b. Because antibiotics remove all gut microbes, leaving a person germ-free and unable to digest food adequately, so the body stores it as fat
    c. Because antibiotics selectively remove “good” bacteria, allowing the “bad” bacteria to thrive
    d. Because antibiotics reduce microbial diversity, which has been linked with increased obesity

10. Studies have show that disruptions to the infant gut microbiome, due to either Caesarean delivery or antibiotic use in infancy, increase risk of which of the following conditions? (select all that apply)
    a. Allergies to harmless environmental factors such as pollen, cat dander, or peanuts
    b. Asthma
    c. Childhood leukemia
    d. Childhood obesity
    e. Acromegaly and gigantism
    f. Autoimmune diseases
